# Supplementary material for: Expression of Basement Membrane Molecules by Wharton Jelly Stem Cells (WJSC) in Full-Term Human Umbilical Cords, Cell Cultures and Microtissues
Source: Cells. 2023 Feb 15;12(4):629. doi: 10.3390/cells12040629 (PMC9954414; doi:10.3390/cells12040629)
Supplement: Supplementary file 1 [file cells-12-00629-s001.zip › cells-2209986-supplementary.pdf]

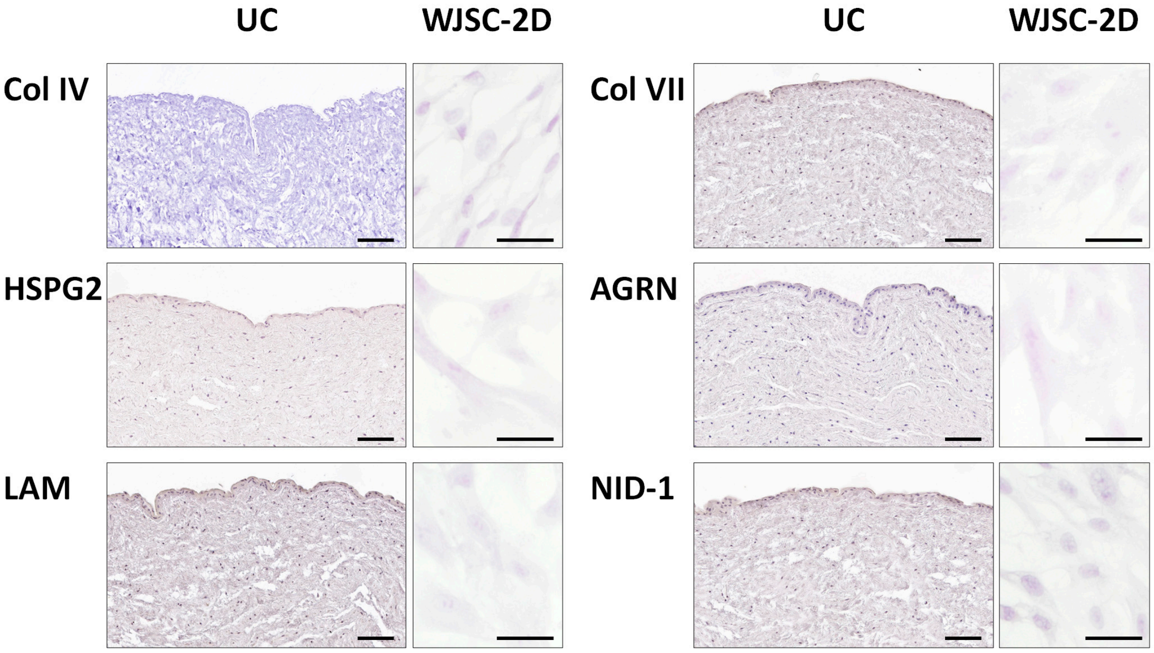

Figure S1. Negative technical controls for immunohistochemical analyses of the basement membrane (BM) molecules [collagen type IV and VII (Col IV) (Col VII), proteoglycans heparan sulfate proteoglycan 2 (HSPG2) and agrin (AGRN) and glycoproteins laminin (LAM) and nidogen-1 (NID-1)] in full-term human umbilical cords (UC) and Wharton's Jelly stem cells under two -dimensional cell culture (WJSC-2D). Images showed negative reaction in amnios basement membrane, cells of UC stroma and WJSC-2D. Scale bar: 100 μm (UC) and 50 μm (WJSC-2D).
